# Complement C1q Interacts With LRP1 Clusters II and IV Through a Site Close but Different From the Binding Site of Its C1r and C1s-Associated Proteases

**DOI:** 10.3389/fimmu.2020.583754

**Published:** 2020-10-21

**Authors:** Guillaume Fouët, Evelyne Gout, Catherine Wicker-Planquart, Isabelle Bally, Camilla De Nardis, Stéphane Dedieu, Anne Chouquet, Christine Gaboriaud, Nicole M. Thielens, Jean-Philippe Kleman, Véronique Rossi

**Affiliations:** ^1^Université Grenoble Alpes, CNRS, CEA, IBS, Grenoble, France; ^2^Bijvoet Center for Biomolecular Research, Department of Chemistry, Faculty of Science, Utrecht University, Utrecht, Netherlands; ^3^Université de Reims Champagne-Ardenne, UMR CNRS 7369 MEDyC, Reims, France

**Keywords:** complement C1q, scavenger receptor, LRP1, CD91, interaction

## Abstract

LRP1 is a large endocytic modular receptor that plays a crucial role in the scavenging of apoptotic material through binding to pattern-recognition molecules. It is a membrane anchored receptor of the LDL receptor family with 4 extracellular clusters of ligand binding modules called cysteine rich complement-type repeats that are involved in the interaction of LRP1 with its numerous ligands. Complement C1q was shown to interact with LRP1 and to be implicated in the phagocytosis of apoptotic cells. The present work aimed at exploring how these two large molecules interact at the molecular level using a dissection strategy. For that purpose, recombinant LRP1 clusters II, III and IV were produced in mammalian HEK293F cells and their binding properties were investigated. Clusters II and IV were found to interact specifically and efficiently with C1q with K*_Ds_* in the nanomolar range. The use of truncated C1q fragments and recombinant mutated C1q allowed to localize more precisely the binding site for LRP1 on the collagen-like regions of C1q (CLRs), nearby the site that is implicated in the interaction with the cognate protease tetramer C1r2s2. This site could be a common anchorage for other ligands of C1q CLRs such as sulfated proteoglycans and Complement receptor type 1. The use of a cellular model, consisting in CHO LRP1-null cells transfected with full-length LRP1 or a cluster IV minireceptor (mini IV) confirmed that mini IV interacts with C1q at the cell membrane as well as full-length LRP1. Further cellular interaction studies finally highlighted that mini IV can endorse the full-length LRP1 binding efficiency for apoptotic cells and that C1q has no impact on this interaction.

## Introduction

**Figure 1:**
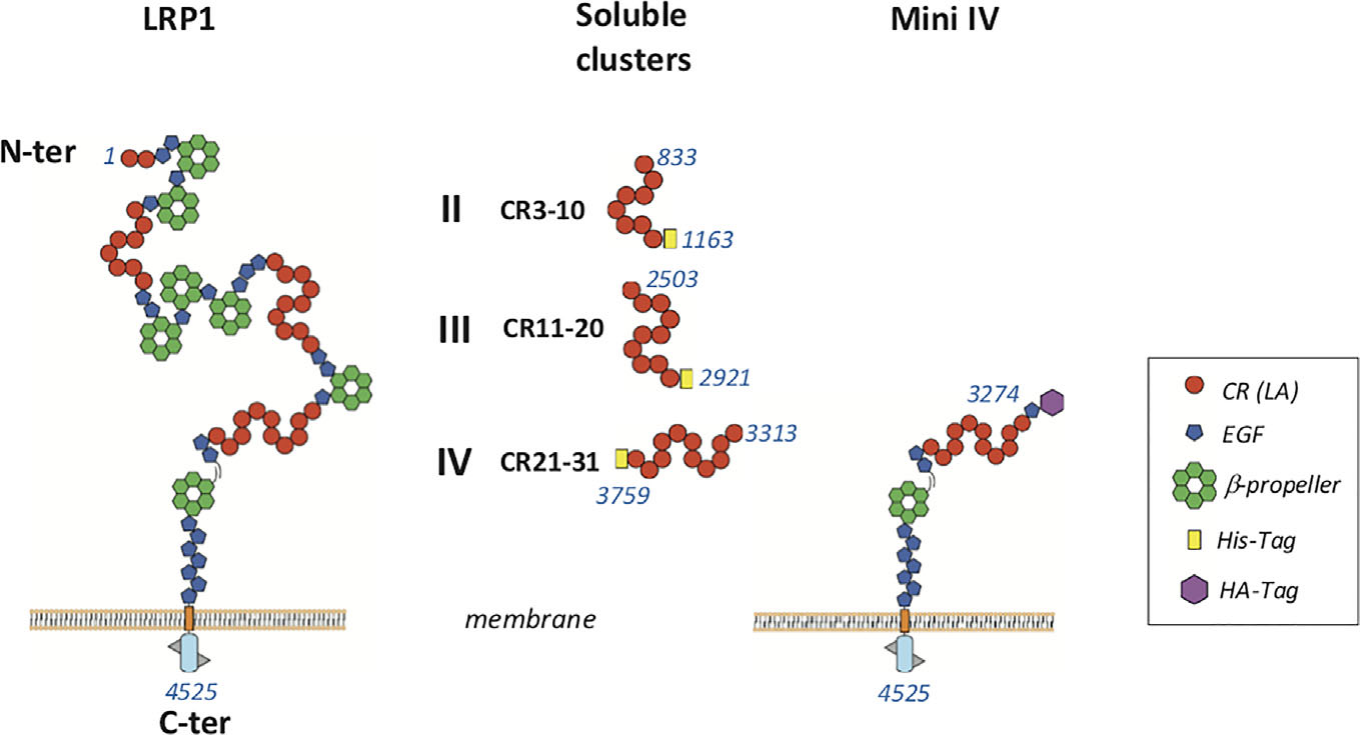
Schematic representation of LRP1 fragments used in this study LRP1 clusters II, III and IV were produced in 293-F mammalian cells with a His-Tag at the C-terminal extremity. Membrane full-length LRP1 and minireceptor IV (mini IV) were used for cellular interaction studies. CR, complement repeat; LA, LDL receptor class A; HA, hemaglutinin; EGF, epidermal growth factor. Amino acid numbering of mature LRP1 is indicated in blue.

C1q is a defense collagen that is known for decades for its implication in the elimination of pathogens or altered-self bodies through the classical cascade of complement. In this context C1q recognizes targets and triggers the complement cascade through activation of an associated protease tetramer C1r2s2. Nowadays, a large body of research also highlights some widely diverse, non-complement related functions of C1q. As examples, C1q can act as an opsonin bridging targets and membrane receptors, C1q is implicated in the modulation of immune cells differentiation and it has an essential role in the enhancement of apoptotic cells phagocytosis (1–3). C1q is a 450 kDa protein assembled from three different polypeptide chains into six stems forming a bouquet like scaffold. C1q exposes six identical globular heads (GR) on one end, extending in six collagen stems (Collagen-Like Regions, CLR) that associate into a bundle on the other end of the molecule. This particular structural arrangement is providing a wide diversity in C1q functions, with the globular heads recognizing targets that for most of them will trigger the classical complement cascade whereas the collagen regions are implicated in other non-complement functions. Removal of apoptotic cells has been described to involve a ternary complex on phagocytic cells that is composed of LRP1, a membrane scavenger receptor belonging to the LDL receptor family, and two soluble proteins, calreticulin (CRT) and defense collagens such as MBL, SP-A and SP-D, or C1q. The implication in efferocytosis of such a membrane complex is nevertheless controversial, such as its molecular arrangement. Some studies describe the beneficial C1q-dependent uptake of apoptotic cells through LRP1/CRT interaction (4, 5), but it also appeared lately, using LRP1 deficient macrophages, that LRP1 is not required in macrophage-mediated C1q-dependent phagocytosis (6). Moreover, it was also shown that C1q interacts in a binary way with LRP1 without the need of CRT (7). LRP1 is a large 600 kDa endocytic receptor that participates in several biological pathways and plays prominent role in endocytosis of a large number of unrelated ligands. It is the largest member of the scavenger receptor family with an extracellular polypeptide extension composed of numerous structurally homologous modules of three types, EGF repeats, β-propeller domains and cysteine-rich calcium dependent complement type repeats called CR or LA modules (**Figure 1**). Four different clusters, I, II, III and IV each composed of respectively 2, 8, 10 and 11 consecutive CR modules are the binding platforms for LRP1 extracellular ligands (8). Clusters II and IV are the targets for most of LRP1 ligands and display only minor differences in binding kinetics whereas few have been described for cluster III (9). When processed inside the cells, LRP1 is associated with a chaperone of 39 kDa called receptor-associated protein (RAP), that binds the three clusters (II, III and IV), and is then eliminated when mature LRP1 becomes exposed outside the cell membrane (10). Extensive studies on RAP binding to LRP1 and dissection of other ligand interactions highlight a common binding strategy of LRP1 CR modules to LRP1 ligands, including a calcium-dependent mode of electrostatic recognition, together with avidity effects resulting from the use of multiple sites. Ligand binding appears to involve the docking of two or more lysine residues into acidic pockets located within CR modules of the receptor referred as “acidic necklace” (11). There are still incomplete data about C1q interaction with LRP1. Duus et al. showed that C1q interacts with LRP1 in the absence of CRT, and that the binding interferes with interaction of ligands of both clusters II and IV (7). In this work we aimed at going further in deciphering this interaction using soluble and membrane clusters of LRP1. We provide evidence that C1q interacts specifically with clusters II and IV at a RAP-competing binding site. We also highlight that cluster IV plays a central role in both C1q and apoptotic cells binding. On C1q, this interaction involves mainly C1q CLR and mobilizes basic residues that are close but different from the protease C1r2s2 binding site. This site could constitute a common “anchor station” shared by other C1q ligands such as sulfated proteoglycans and CR1.

## Materials and Methods

### Proteins, Cells, and Reagents

C1q was purified from human serum and quantified as described (12). Human serum was obtained from the Etablissement Français du Sang (EFS) Rhône-Alpes (agreement number 14-1940 regarding its use in research). C1q collagen stalks (CLR) and C1q globular heads (GR) were prepared according to Tacnet-Delorme et al. (13). The recombinant protease tetramer C1r2s2 was produced and purified according to Bally et al. (14). Full-length LRP1 (soluble LRP1) was produced and purified according to De Nardis et al. (15). Recombinant C1q and C1q mutant LysA59Ala/LysB61Ala/LysC58Ala were produced and purified as described in Bally et al. (16). For protein quantification, Mw and A_1%, 1 cm_ were respectively for C1q (459,300; 6.8) (12), CLR (189,900; 2.1), GR (48,000; 9.3) (17), C1r2s2 (330,000; 13.5) (18). Oligonucleotides were from Eurogentec. Restriction and modification enzymes were from New England Biolabs.

Full-length LRP1 Myc DDK clone (RC218369) was purchased from Origene. The pET22B-RAP plasmid was kindly provided by Søren Moestrup, Aahrus University, Denmark. pcDNA3.1 mini IV HA-tag was cloned as previously indicated (19).

### Cloning of LRP1 Clusters II, III, and IV in pcDNA3.1 for Soluble Expression

The DNA sequence encoding the signal peptide for human LRP1 was inserted in pcDNA3.1/Neo by site directed mutagenesis using the QuickChange II XL kit (Agilent Technologies) according to an optimized procedure (20). A nucleotide sequence encoding the four amino acids, AIDA, located after the signal peptide cleavage site of LRP1 (10) plus a *BamH*I restriction site were introduced on the 3′-end of the sequence coding for the signal peptide in order to subclone soluble cluster sequences between *BamH*I and *Xho*I sites.

The cDNA for each cluster II, III and IV corresponding respectively to the mature LRP1 amino acid fragments 833-1163 (II), 2503-2921 (III), and 3313-3759 (IV) with a supplementary sequence coding for a 7 (cluster III) or 8 His-TAG (cluster II and IV) on the 5′ end, was generated by PCR amplification of full-length LRP1 Myc DDK and cloned in pcDNA3.1/LRP1 signal peptide vector as mentioned above.

### Production of LRP1 Soluble Clusters II, III, and IV in HEK293F Cells and Purification

pcDNA 3.1 plasmids coding for each cluster II, III and IV were transfected in Freestyle HEK293F (293-F) cells using 293fectin, according to the manufacturer’s protocol (Invitrogen) and stabilized by G418 selection (400 μg/ml). Around 500 ml of expression medium were harvested and submitted to two-step purification. First, the medium was dialyzed in 20 mM Tris, 150 mM NaCl, pH 7.5 and loaded on a HiTrap™ Chelating HP column (5 ml, GE Healthcare Life sciences). The fractions containing the clusters were then concentrated/diluted in 50 mM sodium acetate, 50 mM NaCl, 10 mM EDTA, pH 6 before loading on an anion exchange column Mono Q^®^ 5/50 GL (GE Healthcare Life sciences) and elution was achieved through a NaCl gradient (50 - 500 mM). Concentration was finally carried out to reach around 0.5 mg/ml and the buffer changed into 20 mM Tris, 150 mM NaCl, pH 7.5.

The concentration of the purified soluble LRP1 clusters was estimated using the absorption coefficient A_1%, 1 cm_ at 280 nm calculated using the PROTPARAM program on the Expasy server, and an experimental molecular weight determined by MALDI mass spectrometry. Their A_1%, 1 cm_ at 280 nm and molecular weight were respectively 11.78 and 50,372 for cluster II, 9.96 and 63,734 for cluster III and 10.16 and 62,528 for cluster IV.

Mass spectrometry analyses were performed on a Matrix Assisted Laser Desorption Ionization-Time of Flight (MALDI-TOF) mass spectrometer (Autoflex, Bruker Daltonics), operated in linear positive mode. The proteins (1 mg/ml) were diluted 1:2 to 1:10 in SA matrix [sinapinic acid (Sigma-Aldrich) 10 mg/ml in acetonitrile/water/trifluoroacetic acid [50/50/0.1 (v/v/v)] and 2 μL were deposited directly on the target.

### RAP Expression and Purification

RAP was overexpressed by pET22B-RAP transformed *Escherichia coli* BL21(DE3) using conventional IPTG induction (1 mM) in LB medium for 3 h at 37°C. Bacteria were lysed by sonication in 100 mM NaCl, 20 mM Tris, 10 mM MgCl_2_, pH 8.5 supplemented with Complete^®^ protease inhibitor cocktail (Roche Diagnostics). The lysate was then purified by nickel-affinity chromatography (His-select, Sigma-Aldrich) followed by gel filtration on Superdex^®^S75 10/300 (GE Healthcare Life sciences). Purification was performed in 100 mM NaCl, 20 mM Tris, pH 8.0. For concentration determination, a molecular mass of 36,440 Da corresponding to the RAP amino acid sequence preceded by a thrombin cleavage site and a 6His-Tag (MHHHHHHLVPRGS … Y) and A_1%, 1 cm_ at 280 nm of 9.26 were calculated by the PROTPARAM program.

### Soluble LRP1 Clusters and RAP Interaction Experiments by SPR

For all surface plasmon resonance experiments, protein ligands were immobilized on CM5 sensor chips using the amine coupling chemistry according to the manufacturer’s instructions (GE Healthcare). Immobilizations were performed at 10 µl/min in 10 mM HEPES, 150 mM NaCl, 3 mM EDTA, 0.005%, surfactant P20, pH 7.4 (HBSEP, for BIAcore 3000) or the same buffer supplemented with 0.05% surfactant P20 (HBSEP+, for T200 instrument). Regeneration of the surfaces was achieved by 15 μL injections of 1 M NaCl, 10 mM EDTA.

**RAP interaction with full-length LRP1 and clusters II, III, and IV** was determined on a BIAcore 3000 instrument (GE Healthcare). Full-length LRP1 was diluted at 50 µg/ml in 10 mM sodium acetate pH 3.5 to get an immobilization level of 7,400 RU. Clusters were diluted at 10 µg/ml in 10 mM sodium acetate pH 4.0 (clusters II and III) and 4.5 (cluster IV). For interaction measurements, RAP (ranging from 0.25 to 16 nM) was injected over immobilized clusters II (2,967 RU), III (3,460 RU) and IV (3,396 RU) in 50 mM triethanolamine-HCl (TEA), 150 mM NaCl, 1 mM CaCl_2_, 0.005% P20, pH 7.4 at 20 µl/min. Kinetic data were analyzed by global fitting to a 1:1 Langmuir binding model of both the association and dissociation phases using the BIAevaluation 3.2 software (GE Healthcare).

**Interaction of soluble clusters II, III, and IV with immobilized C1q** was also performed on a BIAcore 3000. For that, serum C1q was diluted in 10 mM sodium acetate, pH 5.5 at 23 μg/ml and injected over the CM5 chip to get the immobilization level of 17,500 RU. Cluster II, III and IV (500 nM) interaction was done in 50 mM TEA, 150 mM NaCl, 1 mM CaCl_2_, 0.005% P20 pH 7.4 at 20 µl/min.

**Serum C1q, C1q GR, and CLR binding to immobilized clusters II and IV and C1r2s2 competition** was performed on a T200 instrument. Both clusters were diluted at 50 µg/ml in 10 mM sodium acetate, pH 4 for immobilization. The interaction of C1q or CLR or GR on immobilized LRP1 cluster II (1,230 RU) or cluster IV (1,270 RU) was measured in 50 mM Tris, 150 mM NaCl, 2 mM CaCl_2_, 0.05% P20, pH 7.4 at 30 µl/min with association and dissociation of 180 s. For C1r2s2 competition, C1q was pre-incubated (15 min at 25°C) with C1r2s2 before injection. The equilibrium dissociation constants (*K*_D_) for serum C1q binding to clusters II and IV were determined by injection of concentrations ranging from 0.125 nM to 8 nM. The *K*_Ds_ were calculated from measured binding levels at equilibrium (Req) by fitting plots of Req versus concentration using steady state analysis (Biaevaluation software).

**Determination of the *K*_Ds_ for the interaction of clusters II and IV with immobilized serum C1q, rC1q WT, and rC1q ABC** was performed on a T200 instrument on immobilized serum C1q (14,000 RU), recombinant C1q (11,000 RU) and C1q mutant LysA59Ala/LysB61Ala/LysC58Ala (rC1qABC, 12,300 RU). All C1q samples were diluted in 10 mM sodium acetate, pH 5. The interaction of clusters II and IV was measured by injection of indicated concentrations (see **Figure 7**) in 50 mM Tris, 150 mM NaCl, 2 mM CaCl_2_, 0.05% P20, pH 7.4, for 180 s at 30 µl/min. The equilibrium dissociation constants (*K*_D_) were calculated as mentioned above for serum C1q interaction with immobilized clusters.

### Transfection and Expression of Full-Length LRP1 and Mini IV Receptor in LRP1-Null CHO Cells

#### Cell Culture and Culture Conditions

LRP1-deficient CHO cells called in this study CHO-null cells (21) were obtained from Kanekiyo Takahisa from the Department of Neuroscience, Mayo Clinic, Jacksonville, Florida, USA. Unless otherwise stated, all reagents are from Gibco^®^. CHO-null and Jurkat cells were respectively cultured in DMEM-F12 (Dulbecco’s modified Eagle’s medium), or in RPMI (Roswell Park Memorial Institute medium), supplemented with 10% (v/v) Fetal Bovine Serum (FBS) at 37°C with an humidified atmosphere and 5% CO_2_. For CHO clones expressing LRP1 receptors (full-length or mini IV), the media were supplemented with G418 (geneticin sulfate) at 400 µg/ml.

#### Transfection of Full-Length and Mini IV LRP1 Receptors in CHO-Null Cells and Analysis of Receptors Expression

Plasmid DNAs were transfected into CHO-null cells by lipofectamine 2000 following manufacturer’s instructions (Invitrogen™). Briefly, 24 h before transfection, cells were plated in 35 mm dishes (or 6-well plates) at 0.5 10^6^ cells per dish, in 1.5 ml of culture medium without G418, to reach 70% confluency at transfection. Four µg of plasmid DNA and 10 µl of lipofectamine 2000 were separately diluted in 250 µl of OptiMEM and mixed. After 15 min incubation, the mix was added to each well, and the cells were further incubated for 72 h at 37°C before adding 400 µg/ml of G418 for selection.

Monoclonal cell populations were isolated and amplified after a series of 3 limit dilutions. Briefly, transfected cells were counted and diluted to inoculate unique cells into a 96-well plate. After 10 to 12 days in culture, full-length LRP1 or cluster IV mini-receptor expression and cell population homogeneity were tested by flow cytometry and immunofluorescence microscopy as detailed below.

### Flow Cytometry

Adherent CHO-K1 transfected cells were recovered using Gibco^®^ Versene buffer and further washed in PBS (Phosphate-Buffered Saline) supplemented with 1% BSA (Bovine Serum Albumin; Sigma-Aldrich). For each sample, 1 × 10^6^ cells were incubated on ice for 45 min in 100 µl of anti-CD91-PE (BD Biosciences; dilution 1/5 in PBS 1% BSA) for full-length LRP1 receptor, or 100 µl of anti-HA-PE (Miltenyi biotech, Bergish Gladbach, Germany; dilution 1/20 in PBS 1% BSA) for mini IV LRP1 truncated receptor. Cells were then washed twice in PBS 1% BSA before analysis (MACSQuant VYB flow cytometer - Miltenyi Biotech, Bergish Gladbach, Germany) using the 561 nm excitation and 586(15) nm emission channel (Y1). PE positive populations were estimated after forward scatter (FSC) and side scatter (SSC) gating on the cells. For each condition, at least 20,000 events were analyzed.

### Immunofluorescence Microscopy

Naïve or transfected CHO-null cells cultured on coverslip were fixed in 4% (w/v) paraformaldehyde (PFA) and processed for immunofluorescence using LRP1 full-length or mini IV receptors labeling respectively with anti-CD91-PE (BD Biosciences; dilution 1/5 in PBS 1% BSA) or anti-HA-PE (Miltenyi biotech, dilution 1/20 in PBS 1% BSA). Alternatively, secondary labeling with Cy3-conjugated anti-mouse antibody (dilution 1/250 in PBS 1% BSA) of anti-CD91 (BD Biosciences; dilution 1/250 in PBS 1% BSA) and of anti-HA (anti-HA.11 Biolegend 1/1000 in PBS 1% BSA) were used for LRP1 and mini IV receptors, respectively. Coverslips were mounted on slides using Vectashield with DAPI (Vector laboratory). Pictures were acquired with IQ software (Andor™), using a spinning disk confocal microscope (Yokogawa CSU-X1–IX81 Olympus) with an iXon EMCCD camera (Andor™), and the appropriate channels for PE and DAPI visualization.

### Interaction of C1q with Transfected Cells

CHO-null, or expressing either full-length LRP1 or mini IV LRP1 truncated receptors were detached using Versene, washed once in PBS 1% BSA and resuspended in the same buffer. For each condition, 1 × 10^6^ cells were incubated 30 min on ice with 8 µg of C1q in 100 µl PBS 1% BSA. After 2 washes with 1 ml of PBS 1% BSA, bound C1q was detected by immunostaining using a monoclonal anti C1q antibody (mAB A201 Quidel Corporation, dilution 1/100 in PBS 1% BSA) for 45 min on ice, followed by Cy3-conjugated goat anti-mouse antibody (dilution 1/250 in PBS 1% BSA for 30 min on ice). Flow cytometry analyses were performed on a MACSQuant VYB cytometer as already described, using the 561/586(15) nm Y1 channel for Cy3.

### Interaction of Jurkat Cells With LRP1 Receptors Expressing CHO Cells and C1q Impact Determination

The cell-cell interaction assay was performed using flow cytometry of differentially labeled Jurkat and CHO-null cells expressing either none, full-length LRP1, or mini IV receptors. In brief, 24 h before the assay, cells were harvested and labeled using PKH26 for CHO clones or PKH67 for Jurkat cells, according to manufacturer instructions (Sigma-Aldrich). The labeling reaction was stopped after 5 min incubation in the dark by adding pure FBS. After washing, CHO control and clones expressing LRP1 constructs were plated at 2 × 10^5^ cells per ml culture medium in 12-well plate. PKH67 labeled Jurkat cells were resuspended in complete RPMI medium at 1 × 10^6^ cells per ml and when required, apoptosis was induced by UVB irradiation at 312 nm for 5 min (500 mJ/cm^2^) in 60* mm* cell culture dish (5 ml/dish). 16 h after labeling healthy or late apoptotic Jurkat cells were centrifuged and counted. This treatment yields around 74% of apoptotic Jurkat cells. When required, Jurkat cells (2 × 10^6^ cells) were incubated with 15 µg of C1q in 100 µl DPBS (Dulbecco’s PBS in the presence of calcium and magnesium, 3% BSA) for 1 h on ice. For interaction tests, 2 × 10^6^ PKH67 labeled Jurkat cells were washed in 1 ml DPBS, resuspended in 1 ml of complete DMEM-F12 and added to the monolayer of CHO-K1 cells labeled with PKH26 in the 12-well plates. After incubation at 37°C (typically 2 h), the CHO-K1 cells in a monolayer were washed with 1 ml DPBS to remove the non-attached Jurkat cells. PKH26-labeled CHO-K1 cells decorated with PKH67 labeled Jurkat cell were recovered by gentle scraping or using 100 ml of trypsin solution (Trypsin EDTA, Gibco^®^) before analysis by flow cytometry as described above, using the 561/586(15) nm Y1 channel for PKH26 and the 488/525(50) nm B1 channel for PKH67.

## Results

### Production and Purification of Soluble LRP1 Clusters From Mammalian Cells in Culture

**Figure 2:**
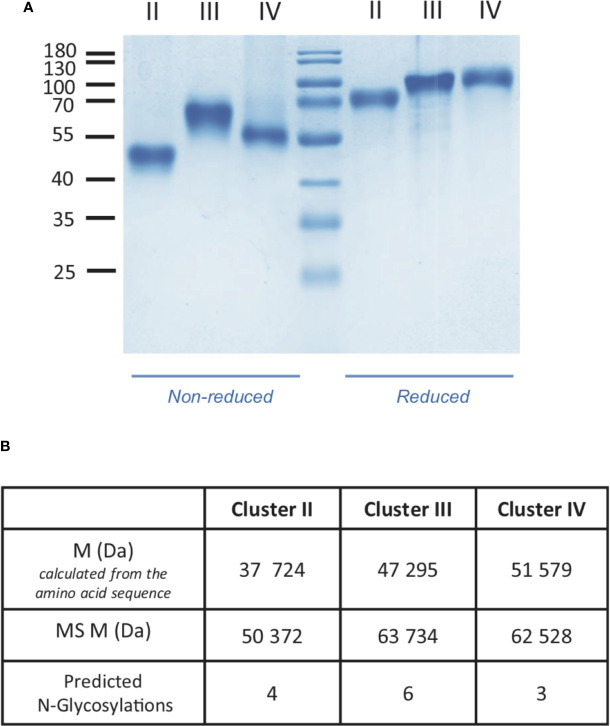
Characterization of LRP1 clusters. **(A)** SDS-PAGE analysis of LRP1 clusters. Clusters II, III and IV in non-reducing (left) and reducing conditions (right), molecular weight markers are indicated in kDa. **(B)**, MALDI mass spectrometry analysis of LRP1 clusters. M, molecular weight; MS, Mass spectrometry.

Soluble LRP1 clusters II, III, and IV were produced in 293-F cells and purified from the culture supernatant by nickel affinity and anion exchange chromatography as described in *Materials and Methods*. These clusters were designed to get soluble fragments restricted to the CR modules described as functional interacting LRP1 regions. In our study, clusters II, III and IV are therefore respectively composed of 8, 10, and 11 CR modules (CR3-10, CR11-20, and CR21-31) (**Figure 1**). To ensure proper cleavage of the LRP1 signal peptide upon secretion, we chose the strategy of Bu and colleagues (10) with the insertion of 4 amino acids (AIDA), naturally located in full-length LRP1 after the cleavage site of the signal peptide. The resulting protein fragments for each cluster have all 6 additional N-terminal amino acids, AIDA plus a GS introduced by the cloning process, and carry also a C-terminal 7 or 8 His-TAG (8 for II and IV and 7 for III). The amount of cluster secreted in the 293-F culture medium was two to three times higher for cluster III than for clusters II and IV, with purification yields of respectively around 0.25 mg/L (II), 0.90 mg/L (III) and 0.40 mg/L (IV). As suggested by Bu and colleagues (10), attempts to improve the production yield were carried in the presence of co-expressed RAP, but no expression increase could be achieved (*data not shown*). Nevertheless, the purification procedure leads to pure secreted fragments as observed by SDS-PAGE analysis (**Figure 2A**). MALDI mass spectrometry analyses (**Figure 2B**), gave for each cluster a mass increase when compared to the calculated polypeptide mass, resulting from post-translational modifications, such as N-linked oligosaccharides, which is consistent with the apparent molecular weights observed on the gel.

### Validation of RAP and LRP1 Clusters as Tools for Deciphering LRP1 Ligand Interaction Properties

**Figure 3:**
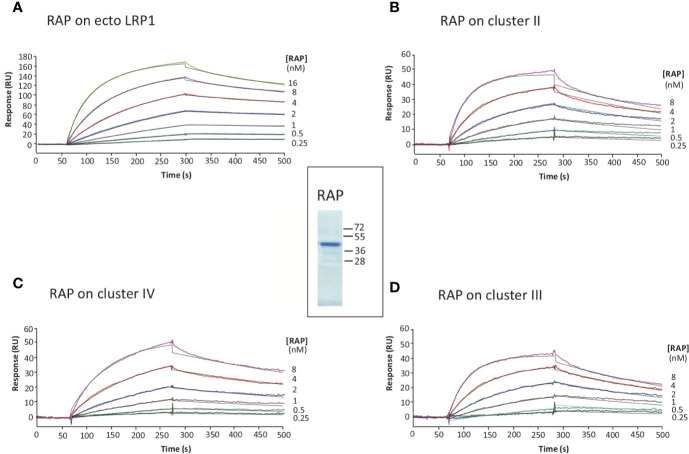
Kinetic analysis of RAP interaction with immobilized full-length LRP1 and clusters II, III and IV. SDS-PAGE of RAP in reducing conditions, molecular weight markers are indicated in kDa (center panel). SPR analysis of RAP interaction with full-length LRP1: RAP was injected at indicated concentrations over immobilized full-length LRP1 (**A**, 7,400 RU), and LRP1 clusters II (**B**, 2,967 RU), III (**D**, 3,460 RU) and IV (**C**, 3,396 RU) in 50 mM triethanolamine-HCl (TEA), 145 mM NaCl, 1 mM CaCl_2_, 0.005% surfactant P20, pH 7.4. The fitting obtained using a Langmuir 1:1 binding model is shown in black lines. Kinetic constants are summarized in **Table 1**.

RAP was expressed in *E. coli* and purified using a two-step chromatography protocol (section 2.4 M&M). The apparent molecular mass of purified RAP observed by SDS-PAGE analysis (**Figure 3**) is as expected around 40 kDa (22, 23). For functional validation, we tested its interaction with soluble full-length LRP1 (also called ecto-LRP1 by De Nardis and colleagues) (15) using surface plasmon resonance. The kinetic constants gave a *K*_D_ value of 0.76 ± 0.08 nM, and association and dissociation constants of respectively 1.41 ± 0.17 × 10^6^ M^−1^ s^−1^ and 1.05 ± 0.07 × 10^−3^ s^−1^ (**Figure 3**, **Table 1**). The interaction of RAP with purified clusters II, III and IV was also investigated and gave *K*_D_ values in the same nanomolar range of respectively 1.09 ± 0.31 nM (II), 1.06 ± 0.1 nM (III) and 1.28 ± 0.12 nM (IV) (**Figure 3**, **Table 1**). These results are in agreement with previous studies (24–26) indicating that RAP interaction with LRP1 is strong and that the affinity of RAP for each individual cluster is in the same range. In the present study, RAP interaction allowed the validation of the functional integrity of the expressed LRP1 clusters.

**Table 1:** Kinetic and equilibrium dissociation constants for the binding of RAP to immobilized ecto-LRP1 and clusters II, III, and IV.

	*k*a (M^−1^ s^−1^)	*k*d (s^−1^)	*K*_D_ (nM)	*n^a^*
Immobilized ecto LRP1	1.41 ± 0.17 × 10^6^	1.05 ± 0.07 × 10^−3^	0.76 ± 0.08	4
Immobilized cluster II	2.20 ± 1.42 × 10^6^	1.96 ± 0.86 × 10^−3^	1.09 ± 0.31	2
Immobilized cluster III	2.95 ± 0.37 × 10^6^	3.08 ± 0.09 × 10^−3^	1.06 ± 0.10	2
Immobilized cluster IV	1.18 ± 0.40 × 10^6^	1.47 ± 0.37 × 10^−3^	1.28 ± 0.12	2

Values are expressed as means ± SE.

n^a^ Number of separate experiments.

### The Interaction of C1q With LRP1 Is Involving Clusters II and IV

**Figure 4:**
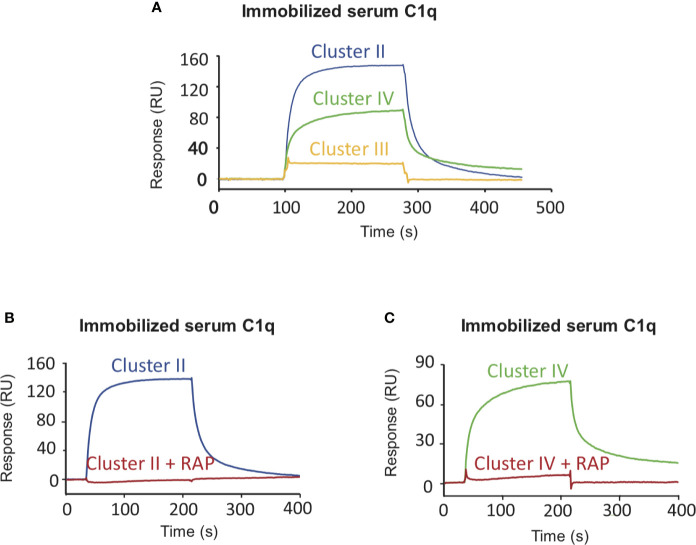
LRP1 clusters II and IV interact with C1q. SPR analysis of LRP1 clusters II (blue curves), III (yellow curves) and IV (green curves) interactions over immobilized C1q, in the presence or absence of RAP. Clusters II, III and IV (500 nM in 50 mM TEA, 150 mM NaCl, 2 mM CaCl2, 0.005% P20, pH 7.4) were injected over immobilized serum C1q (17,500 RU) with or without RAP (500 nM). **(A)** comparison of the interaction curves in the absence of RAP for clusters II, III and IV. **(B, C)** Comparison of the binding of Cluster II **(B)** and Cluster IV **(C)** in the presence or in the absence of RAP.

**Figure 5:**
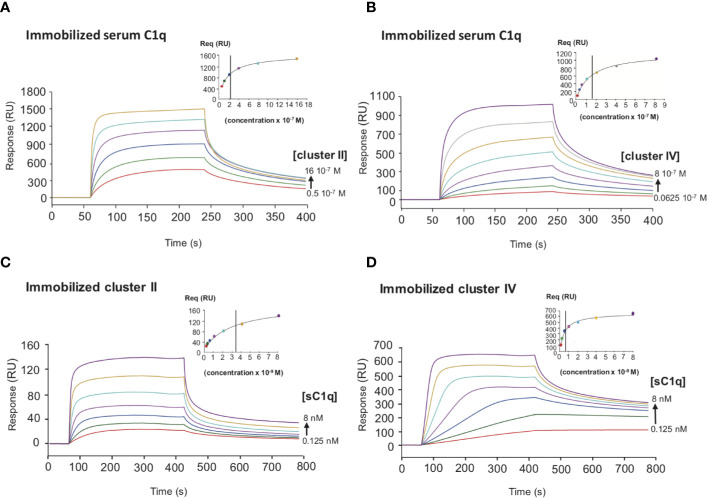
Comparative kinetic analysis of the interaction of serum C1q with LRP1 clusters II and IV. SPR analysis of LRP1 clusters II and IV interactions with serum C1q in two configurations. Top panels, clusters II and IV were injected over immobilized serum C1q (14,000 RU), **(A, B)** respectively. Bottom panels, serum C1q was injected over immobilized clusters II (1,230 RU) and IV (1,270 RU), **(C, D)** respectively. All injection were performed in 50 mM Tris, 150 mM NaCl, 2 mM CaCl_2_, 0.05% P20, pH 7.4. The concentration of soluble ligands is indicated on each curve. The Req versus concentration plots of steady state fittings are shown on the top right of the SPR curves. *K*_D_ values (means ± SD) for each kinetics are summarized in **Table 2**.

Several studies highlighted that most LRP1 ligands are interacting specifically with clusters II and IV (25, 27, 28). The location of LRP1 sites for C1q interaction was one of the questions we aimed at answering first. For that, SPR analyses were performed with clusters II, III and IV injection over immobilized serum C1q. Clusters II and IV interacted similarly with C1q whereas cluster III did not (**Figure 4A**). Moreover, the interaction was efficiently overcome by RAP competition in an equimolar ratio indicating that RAP interaction sites on LRP1 might be shared for C1q interaction or be positioned in a neighbouring region close enough to get competition (**Figures 4B, C**). SPR kinetic analysis shown in **Figure 5**, also pointed out that serum C1q has affinities for both clusters II and IV that are in the same range. With immobilized serum C1q, clusters II or IV interaction had both *K*_Ds_ in a sub-micromolar range (**Figure 5**, **Table 2**). Interestingly, in the reverse configuration, when serum C1q was injected over immobilized clusters II and IV they had an affinity increase of around 100 fold with respectively 3.49 ± 0.45 nM for cluster II and 0.69 ± 0.1 nM for cluster IV (**Figure 5**, **Table 2**). This increased affinity when the clusters are immobilized compared to the reverse orientation might be explained by the known avidity of C1q for surface bound ligands. From these results, even though in the same range, the affinities of C1q for cluster IV appeared to be higher than for cluster II.

**Table 2:** Equilibrium dissociation constants for the binding of clusters II and IV to C1q.

Immobilized protein	Injected protein	*K*_D_ (M)
cluster II	sC1q	3.49 ± 0.45 × 10^−9^
cluster IV	sC1q	0.69 ± 0.11 × 10^−9^
sC1q	cluster II	2.75 ± 0.55 × 10^−7^
sC1q	cluster IV	1.58 ± 0.02 × 10^−7^
rC1q WT	cluster II	3.18 ± 0.67 × 10^−7^
rC1q WT	cluster IV	1.83 ± 0.02 × 10^−7^
rC1q ABC	cluster II	5.14 ± 0.55 × 10^−7^
rC1q ABC	cluster IV	2.22 ± 0.11 × 10^−7^

Values are expressed as means ± SE of two separate experiments.

### The Interaction Site on C1q for LRP1 Clusters II and IV Is Different From the Proteases Binding Site but Is Located in Close Proximity

**Figure 6:**
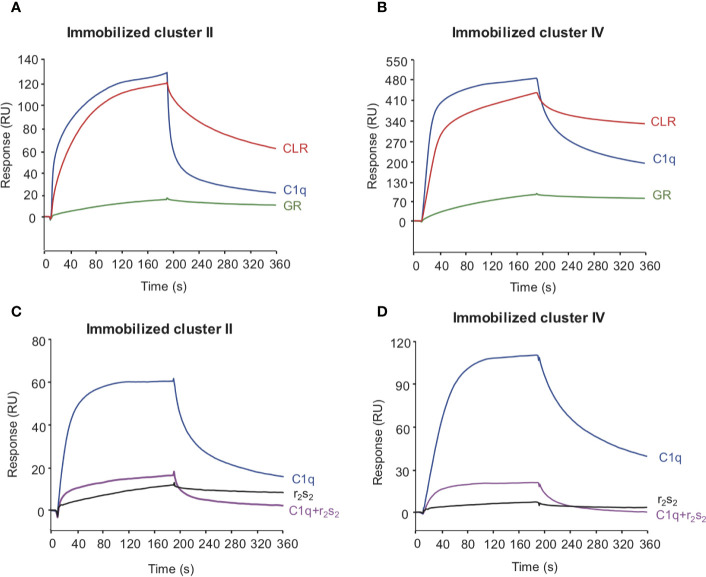
The interaction site of LRP1 clusters II and IV on C1q is located on the collagen stalks at or close to the site interacting with C1r2s2 tetramer. C1q (10 nM, blue curves), CLR (10 nM, red curves) and GR (60 nM, green curves) were injected over immobilized LRP1 cluster II (1,230 RU, **A** panel) or LRP1 cluster IV (1,270 RU, **B** panel), in 50 mM Tris, 150 mM NaCl, 2 mM CaCl_2_, 0.05% P20, pH 7.4. C1q (1 nM) was injected on the same amount of clusters II and IV (bottom **C, D**) with (pink curves) or without (blue curves) C1r2s2 (1 nM). r2s2 was also injected alone (1 nM) as a reference (black curves).

**Figure 7:**
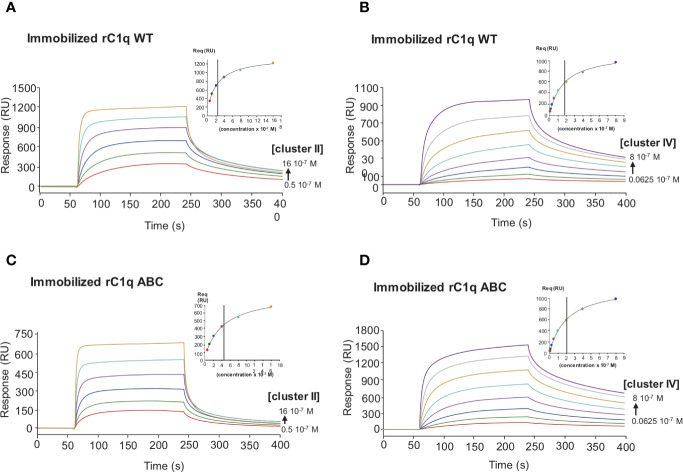
Comparative kinetic analysis of the interaction of LRP1 clusters II and IV with immobilized recombinant C1q variants. LRP1 clusters II (left panels) and IV (right panels) were injected at indicated concentrations over immobilized recombinant C1q (rC1q WT, 11,000 RU, **A**, **B** panels) or C1q mutant LysA59Ala/LysB61Ala/LysC58Ala (rC1q ABC, 12,300 RU, **C**, **D** panels) in 50 mM Tris, 150 mM NaCl, 2 mM CaCl_2_, 0.05% P20, pH 7.4. The Req versus concentration plots of steady state fittings are shown on the top right of the SPR curves. *K*_D_ values (means ± SD) for each kinetics are summarized in **Table 2**.

To decipher the interaction of LRP1 with C1q, both clusters II and IV were first tested for their binding to two separate regions, the globular heads (GR) or collagen regions (CLR) obtained from purified serum C1q. The results of **Figure 6** indicate that the CLRs contribute to almost all the interaction of C1q with both clusters, with a small contribution from the GRs. That observation then raised the question of defining more precisely the location of the binding site of LRP1 on the CLR. For that purpose, an equimolar amount of C1r2s2, whose binding site has been previously identified on C1q CLR (16) was added to C1q prior to its injection on immobilized clusters. The large decrease observed for C1q interaction with cluster II and cluster IV in the presence of C1r2s2 indicates that the protease tetramer competes with LRP1 for binding to C1q (**Figure 6**). The remaining signal can be explained by the contribution of the GRs that are not interacting with the protease tetramer and therefore remain free for binding. These results suggest that the interaction site of C1q for LRP1 may be the same as the C1q binding site for the serine protease tetramer. To go further in the location of the site interacting with LRP1, we used a recombinant variant of C1q, carrying mutations of LysA59, LysB61, and LysC58 (called rC1qABC) and devoid of the C1r2s2 binding capacity (16). Unexpectedly, the results of **Figure 7** highlight that wild-type and mutated C1q interact with both clusters II and IV in the same manner. Indeed, kinetic analyses for all C1q proteins tested yielded affinities ranging from 2.75 to 5.14 × 10^−7^ M for cluster II and 1.58 to 2.2 × 10^−7^ M for cluster IV interactions (**Table 2**). Overall, these data reveal that LRP1 binding to C1q involves one or some site(s) located in close proximity but distinct from the tetramer binding site.

### C1q Interacts With Full-Length LRP1 and Mini Receptor IV at the Surface of Transfected CHO Cells

**Figure 8:**
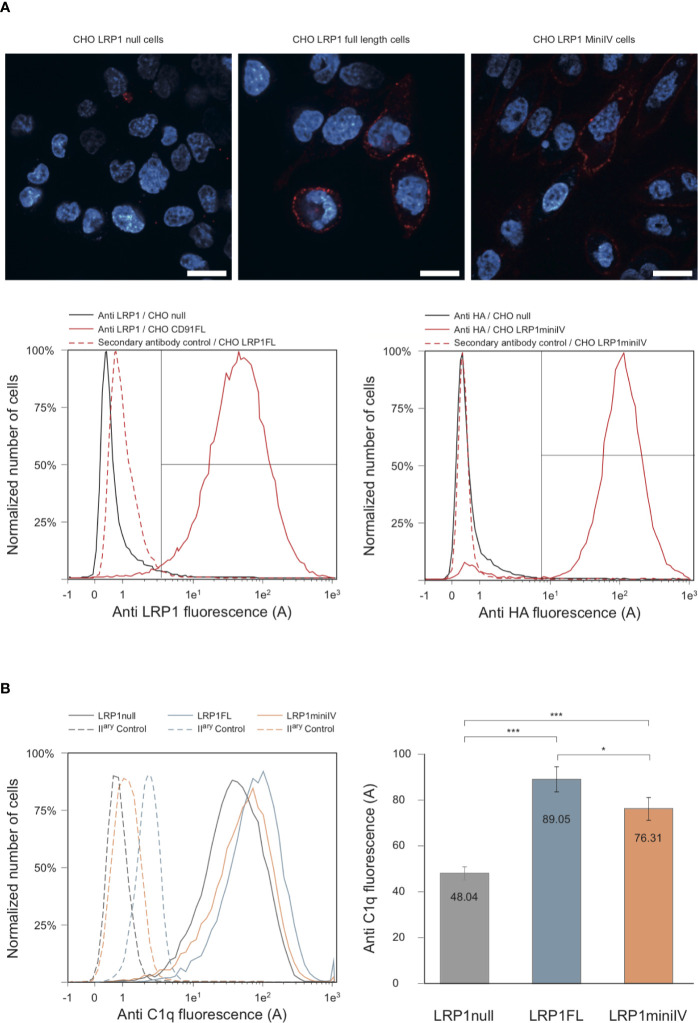
C1q interacts with full-length LRP1 and mini IV at the surface of transfected CHO cells. **(A)** Antibody labeling of LRP1-null CHO cells and CHO cells transfected with full-length LRP1 or mini IV. Top panels, confocal sectioning of LRP1-null, full-length LRP1 and mini IV (red) DAPI counterstained (blue) adherent CHO cells (bars = 20 µm). Bottom panels, histogram overlays of the corresponding cell populations analyzed by flow cytometry. Left, control LRP1-null cells (black) and LRP1 full-length transfected CHO cells (red) labeled with anti LRP1 antibody. Right panel, control LRP1-null CHO cells (black) and mini IV transfected CHO cells (red) labeled with anti HA antibody. Red dotted histograms correspond to secondary antibodies controls. The region for positive cells is shown (brackets) and represents more than 95% of the total cell population. **(B)** Flow cytometric histograms of a typical C1q decoration experiment. Plain lines correspond to C1q binding to LRP1-null cells (grey), full-length LRP1 (blue) and mini IV (red) CHO cells. Dashed lines are the corresponding controls with only secondary antibody only. Right panel, mean C1q fluorescence intensities (corrected from the controls) of six independent experiments. *** Student bilateral unpaired <1 × 10^−4^. * Student bilateral unpaired >4 × 10^−2^.

In order to confirm at the cell surface the results obtained with purified proteins, we expressed full-length LRP1 or the minireceptor IV (mini IV) at the surface of LRP1-null CHO cells and monitored their ability to bind C1q. CHO-null cells were chosen for their non-phagocytic properties in order to get a simple adsorption cellular model (29). LRP1 mini IV consists in the N-terminal truncation of the extracellular portion of LRP1 until the EGF module preceding cluster IV CR21. It therefore contains the full native LRP1 C-terminal region from amino acid 3274 to 4525 (mature protein numbering). A N-terminal HA-Tag has also been added to facilitate LRP1 mini IV immunolabeling (**Figure 1**). The ectopic expression of LRP1 full-length or LRP1 mini IV constructs in LRP1-null CHO cells was confirmed by immunofluorescence (**Figure 8A**). In comparison with the LRP1-null CHO control cells, a clear labeling of the transfected constructs reveals, as expected, the expression of both LRP1 constructs at the plasma membrane. The transiently transfected CHO cells were further selected as described in the Materials and Methods section to obtain enriched populations for LRP1 constructs expression, as probed by flow cytometry (**Figure 8A**). We obtained consistently more than 95% of the cells expressing the receptor constructs and controlled regularly the stable expression of these constructs by flow cytometry to conduct reproducible experiments throughout the present study.

We next used the stable cell populations to study C1q interaction with LRP1 or its truncated counterpart at the cell surface. In brief, LRP1-null, full-length and mini IV cells were preincubated with C1q before being harvested and labeled using anti-C1q antibody. The mean fluorescence intensity of C1q labeling was measured for each cell population and compared to control labeling (**Figure 8B**). The comparison of the measurements from 6 independent experiments, corrected for the secondary antibody only control labeling, underlines the specific increase of C1q binding to LRP1 full-length and mini IV expressing CHO cells in comparison to the mean values obtained on null cells (**Figure 8B**). The binding background observed on null-cells likely arises from the wide variety of C1q cell surface targets (2, 3). Taken together, these results and our data obtained *in vitro* (**Figure 4**) confirm the interaction of C1q with LRP1 and strongly support a role for cluster IV in mediating this interaction both *in vitro* and at the cell surface.

### LRP1 and Cluster IV Are Implicated in the Recognition of Apoptotic Cells

**Figure 9:**
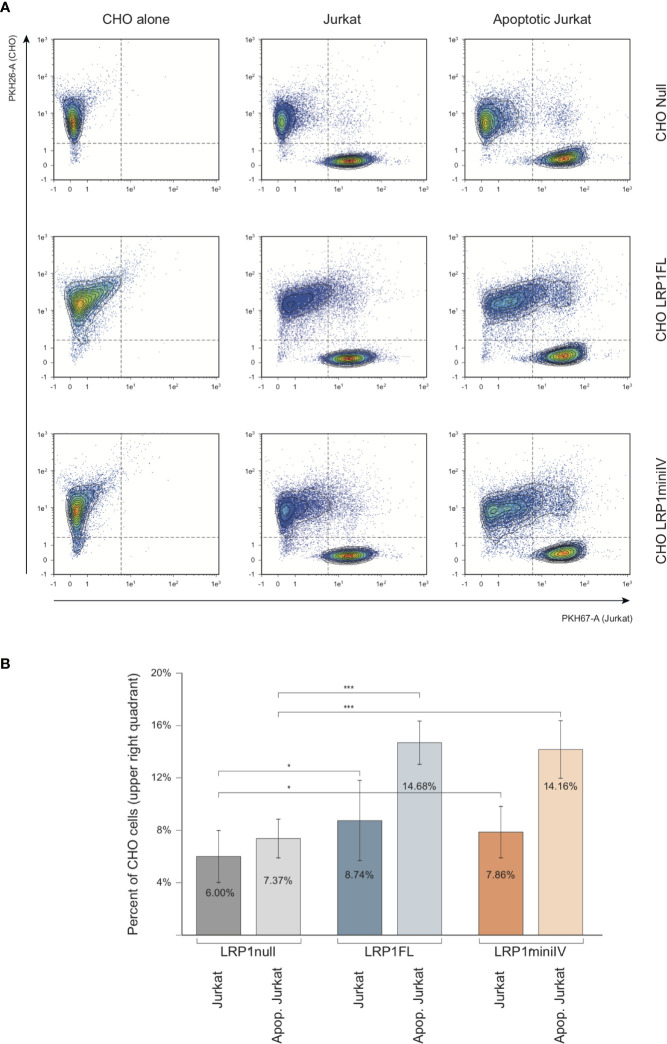
Flow cytometry analysis of the interaction of Jurkat apoptotic cells with LRP1 full-length and mini IV CHO cells. **(A)** Dot plots of the analysis of PKH26 fluorescent CHO cells (red, Y4-A channel) decorated by PKH67 fluorescent Jurkat cells (green, B1-A channel). Decoration efficiency is calculated in the upper quadrant of the region drawn, as the percentage of PKH26 positive events **(B)** Histograms obtained from 13 independent experiments. Student T test relevance is shown above the histograms. *** Student bilateral unpaired <1 × 10^−2^, * Student bilateral unpaired <2 × 10^−1^.

The implication of C1q in apoptotic cell clearance driven by LRP1 has been described in two publications (4, 5). We therefore took benefit of our cell model system to study the differential implication of LRP1 and its cluster IV-containing domain in LRP1-dependent apoptotic cell recognition, using late apoptotic Jurkat cells as baits. Using the membrane-specific vital dyes PKH26 and PKH67 to respectively label CHO and Jurkat cells, respectively. We measured by flow cytometry the binding of PKH67 (Jurkat) to the PKH26 positive cell population (**Figure 9A**). As illustrated in the dot-plot diagram in a representative experiment (**Figure 9A**), the decoration of CHO cells by Jurkat cells is estimated from the percentage of double labeled PKH26 cells among the entire PKH26 population (upper right quadrant; **Figure 9A**). We compared the decoration efficiency of healthy or late apoptotic Jurkat after incubation at 37°C with CHO cells expressing or not the LRP1 full-length (FL) or mini IV constructs. The mean values from 13 independent experiments in each condition and their respective standard deviations are shown in **Figure 9B**. The expression of LRP1 or its truncated form are both enhancing the decoration of CHO cells by late apoptotic Jurkat cells but not by healthy Jurkat cells, suggesting that LRP1 is implicated in the specific recognition of apoptotic cells. A representative specific binding of apoptotic Jurkat cells to LRP1 expressing CHO cells is also shown by time lapse immunofluorescence (**Supplementary Figure S1**). In these experimental conditions as expected from non-phagocytic CHO cells, recognition of the apoptotic Jurkat cells accounts entirely for the adsorption of Jurkat cells as confirmed by a treatment with trypsin before flow cytometry that is reverting the adsorption to the level of the control experiments (**Supplementary Figure S2**).

**Figure 10:**
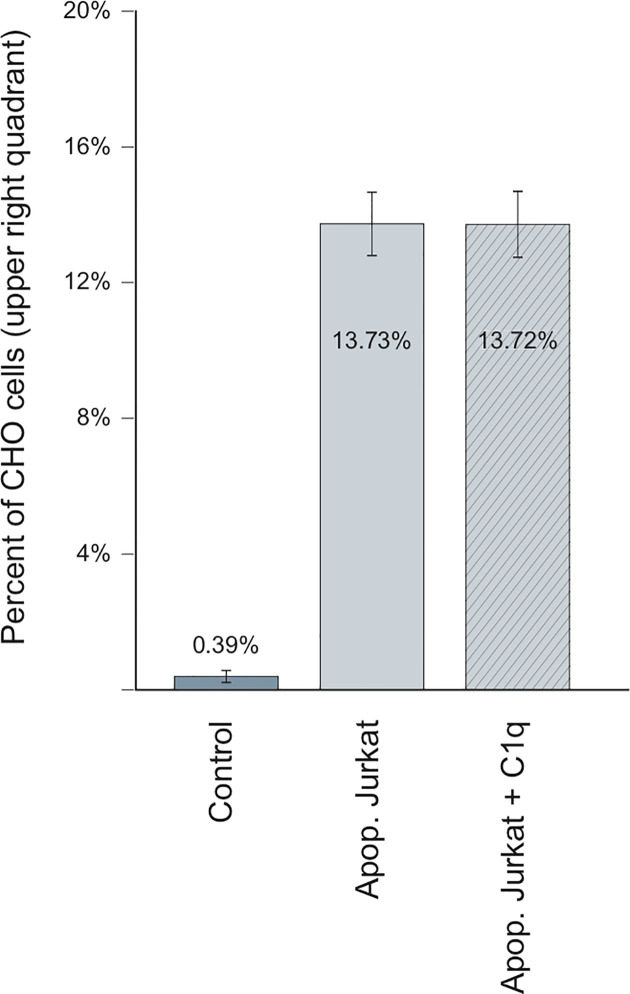
C1q is not implicated in LRP1-dependent adsorption of Jurkat apoptotic cells Flow cytometry analysis of CHO cells stably expressing full-length LRP1, incubated with apoptotic Jurkat cells in the presence (dashed) or absence (plain) of C1q. Histograms representing the percentage of CHO-associated Jurkat cells from 4 independent experiments.

As demonstrated herein, LRP1 expression in CHO-null cells promotes C1q binding (**Figure 8B**). We thus wondered whether C1q could modulate the specific recognition of the late apoptotic Jurkat cells. Using the same experimental set-up, we measured the adsorption of Jurkat cells in the absence or in the presence of 10 µg/ml of C1q purified from serum (**Figure 10**). In these conditions, the recognition of apoptotic cells by CHO cells was not modified by the presence of C1q, even for the cells expressing ectopic full-length LRP1, that have been used for the C1q decoration experiments shown in **Figure 8**. Our data suggest that the specific binding of C1q to LRP1 is not enhancing the LRP1-dependent apoptotic cell recognition in a simplified cellular context.

## Discussion

In this study we demonstrate that the interaction of C1q with LRP1 is involving clusters II and cluster IV, two regions that have been described as interacting with most of LRP1 ligands (25, 27). This complements the study of Duus et al. (7) that proposed from competition experiments with diverse LRP1 ligands that clusters II and IV might be the regions recognized by C1q. We add here undoubtful confirmation, using soluble purified recombinant clusters, that the binding site for C1q is located on the CRs modular region of both clusters II and IV and that this interaction is in the same range of affinity although slightly higher for cluster IV than cluster II (around two to five fold). RAP, used as a tool to validate the functional integrity of the recombinant clusters and for competition for C1q binding to LRP1 *in vitro*, was found to compete very efficiently for the interaction of C1q with the LRP1 clusters. Indeed, an equimolar amount of RAP was sufficient to completely inhibit C1q binding to both clusters. This therefore revealed that, as also observed for other LRP1 ligands, the C1q interaction site might be located on both clusters at the same sites as RAP or involve overlapping sites (25, 27). On C1q side, our CLR and GR fragments binding experiments indicated that the interaction with LRP1 mobilizes mainly the collagen stalks and is involving sites that are buried by the C1r2s2 tetramer binding. Even though C1r2s2 competes with LRP1 for C1q binding in SPR conditions for which LRP1 clusters are immobilized, no LRP1 competition for C1 activation could be observed in a C1q reconstituted serum assay (**Figure S3**). This observation is probably not reflecting what would happen at the cell surface. Indeed, in our complement activation experimental conditions, the competition could only be done with soluble C1q and LRP1 clusters, which is not a typical physiological context. Moreover, the affinity of the C1r2s2 interaction with C1q is stronger than the one of soluble LRP1 clusters which is in favor of the C1 formation in this experimental setting [**Table 2**, (16)].

**Figure 11:**
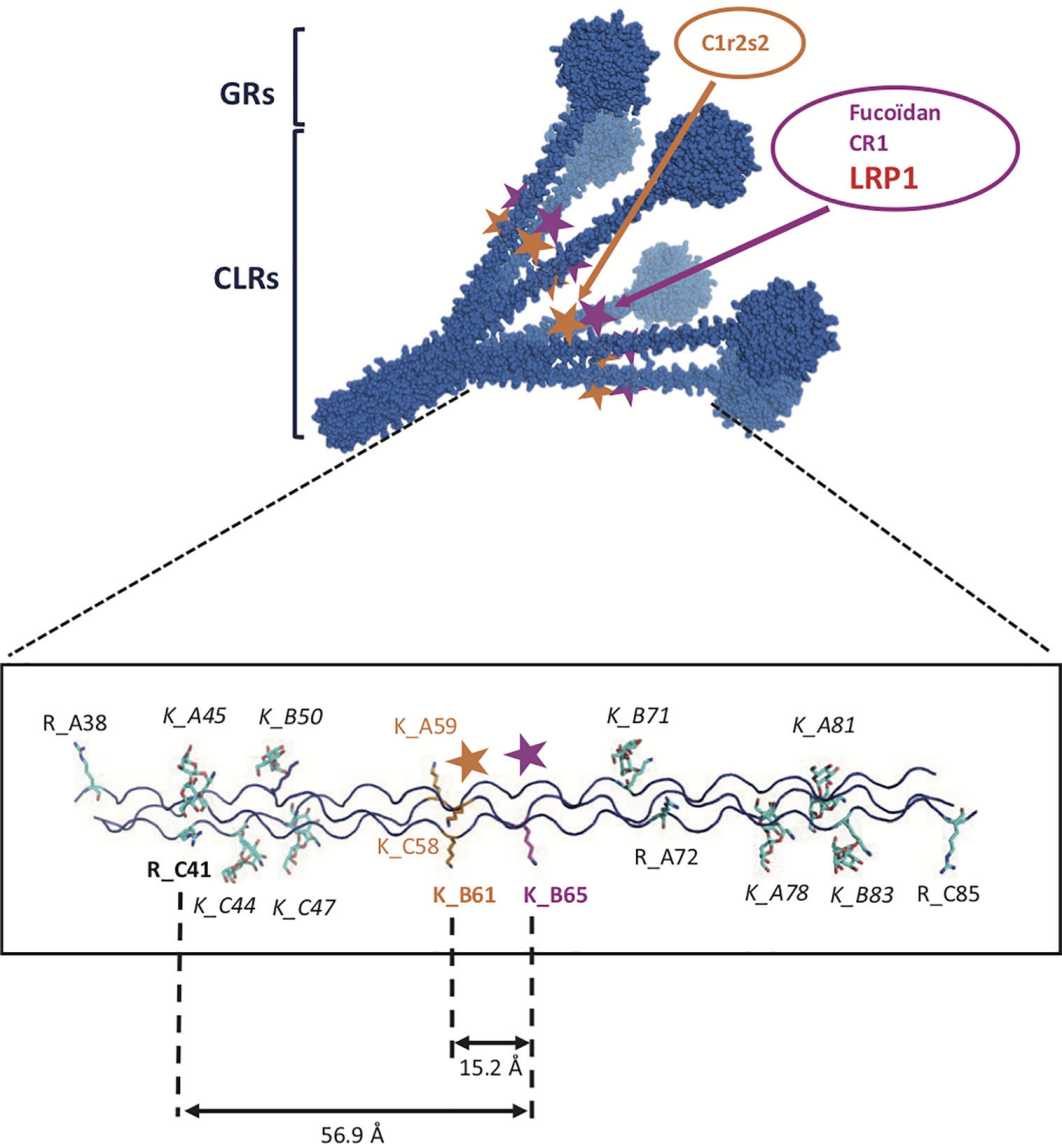
Detailed model of C1q CLRs interaction sites. Upper part: 3-D model of C1q representing a bouquet-like scaffold formed by 6 collagen stalks (CLRs) expending into 6 globular regions (GRs). The two interaction sites for C1r2s2 and for CLR ligands such as LRP1, fucoidan and CR1 are indicated by orange and pink stars, respectively. Bottom part: detailed localization of basic amino acids on one C1q CLR. Lysines or arginines are labeled by R or K letters, followed by the name of the C1q chain, A, B or C and amino acid position. Italics are indicating glycosylated lysines. Bold characters represent residues potentially involved in the binding of LRP1.

A common feature of most of LDL-receptors ligands is their ability to bind to heparin, suggesting the implication of one or more highly positively charged regions in the recognition of the receptor. Indeed, a “Lysine ligand mode” of interaction with tandem CR modules has been described for the binding of LRP1 with its ligands, such as RAP (30), α2Macroglobulin (31), ApoE (32), and factor VIII (33). These interactions are calcium dependent and salt sensitive. LRP1 binding to C1q is inhibited by high salt concentration (0.65 M NaCl) (7) which suggests that it is driven by electrostatic interactions. We also verified that the interaction of C1q with LRP1 clusters II and IV is inhibited by 5 mM EDTA, as expected (*data not shown*). Since C1r2s2 binding to C1q involves ionic, calcium-dependent interactions with C1q lysine residues, one could easily assume these lysines to serve as ligands for LRP1 clusters. To our surprise, a recombinant mutant of C1q lacking these specific lysines still retained its full-binding abilities for both clusters with an affinity in the same nanomolar range as for wild type C1q. These findings suggest that other basic residues in the surrounding could be potential candidates for LRP1 interaction. Contrarily to C1q, in the case of the interaction of LRP1 with MBL, Duus and collaborators observed that the K55 lysine that is implicated in the interaction with the MBL associated proteases MASPs is also responsible for the interaction with LRP1 (34). Moreover, this interaction might involve a single lysine since it is completely abolished by the point mutation K55A of the MBL. The same difference between C1q and MBL behavior was also highlighted in the case of CR1 receptor binding, that was still efficiently interacting with the rC1qABC mutant and no longer with the K55A mutant of the MBL (17, 20). To our knowledge, C1q binds sulfated proteoglycans through its GRs (35) and also even more efficiently through its CLRs (36–38). These studies also evidenced that sulfated proteoglycans inhibit the first step of complement activation by impairing the association of C1r and C1s with C1q. Moreover, through chemical modification using TNBS (2,4,6 trinitrobenzenesulfonate), lysine residues on C1q CLR were shown to be involved in the interaction with fucoidan (39). All together these findings suggest an interaction site on C1q CLR for proteoglycans that could be shared also for LRP1 interaction and that is located nearby the C1r2s2 site. The basic residues in C1q collagen stalks are shown on the model of **Figure 11**. Most of the lysines are modified by O-glycosylation (40), except a proximal lysine 65 of the B C1q chain (K_B65), 15 Å distal to the lysine 61 (K_B61) that is crucial for C1r2s2 binding (16). This K_B65 could be a good candidate for LRP1 interaction. Most of LRP1 ligands have been shown to involve the docking of two or more lysine residues into the acidic pocket of CR modules, which raises the question of a second (or more) basic residue(s) in the interaction of C1q. One possibility could be the interaction with R_C51 which is 56 Å far away from K_B65, on the same molecular face, which remains in the possible range of reported distances between CR acidic pockets (11, 32, 41). Of note even though most of LRP1 ligands involve lysines for interaction with the receptor, arginine can be a possible basic partner of CR module interaction (42). The interaction of LRP1 clusters with C1q at this K_B65 position does not exclude that in the absence of C1r2s2, C1q could interact synergically with both K_B65 and K_B61 and behave differently in our C1q mutant involving other residues, as it was described for RAP multiple lysine mutants (43). It is also not excluded that the single lysine K_B65 could be the only actor of the interaction as it is the case for the K55 of the MBL (34). Taken together our results on LRP1 and similar results obtained in our team for another C1q receptor, CR1 (17), lead us to propose a common site on C1q CLR for receptor interaction involving basic residues distinct but close to the C1r2s2 site.

The comparable affinity of LRP1 ligands for cluster II and IV binding is explained in some studies by a cluster replication that is specific to LRP1. LRP1 is the largest receptor of the LDL-receptor family with multiple biological functions. Since LRP1 such as C1q is a highly flexible protein that interacts with a wide variety of structurally unrelated ligands, in a multivalent manner, it is difficult to imagine a simple model for C1q interaction. Nevertheless, if C1q, like many other ligands, shares affinities for clusters II and IV, it is not evidenced in our study that they interact on both clusters synergically. This might be difficult if we consider that the potential residues for LRP1 interaction are oriented towards the center of the C1q cone (**Figure 11**). Some LRP1 ligands have been described to interact better with cluster IV than with cluster II which is also what we observe in our study (33, 44). From that observation, one could hypothesize that the LRP1 ligands bind first to the cluster that is more distant from the membrane (cluster II) and then interact with cluster IV. Interestingly, MBL and L-ficolin were found to interact with LRP1 (34) and we also localized the interaction site for MBL on cluster II and IV as it is the case for C1q (*data not shown*). Taken all together these data could indicate that the interaction mode of LRP1 could be enlarged to other members of the defense collagen family.

We then aimed at confirming that C1q interaction was also happening at the cell surface using a simple non-phagocytic cellular model. For that purpose, LRP1-null cells were transfected with DNA coding for full-length LRP1 and a mini receptor IV that was chosen preferentially because of its natural location close to the membrane. Our results show that C1q interacts indeed with both LRP1 and minireceptor IV and that the interaction of mini IV receptor is nearly as important as that of full-length LRP1 (85% of the binding compared to LRP1). These results also suggest that other sites might contribute (but to a lesser extent) to LRP1 binding to C1q that could possibly be located on cluster II, the second site identified by our *in vitro* experiments. Since C1q and LRP1 were reported to be important in efferocytosis (1) we naturally addressed the question of apoptotic cells interaction with LRP1 and the implication of C1q in their initial recognition step. Our results indicate that LRP1 full-length and LRP1 mini IV are involved in late apoptotic cells binding in the absence of C1q, with a 100% increase compared to LRP1-null cells. The interaction is increased in the same proportions for full-length and mini IV LRP1 indicating that the binding capacity of the whole LRP1 molecule for late apoptotic cells could be fully endorsed by cluster IV. No LRP1 dependent difference could be observed on the binding of early apoptotic cells (*data not shown*).

The implication of C1q in the removal of apoptotic cells through binding to LRP1 is controversial. While Ogden and Vanvidier et al. (4, 5) observed that a ternary complex including C1q/LRP1 and CRT participates in the phagocytosis of apoptotic cells, Lillis et al. (45) found that the apoptosis enhancement by C1q is independent of LRP1 uptake of apoptotic cells and clearance. In our cell surface model, we focused on the first step of this removing process by looking at the role of C1q in LRP1 adsorption of apoptotic cells and could show that C1q has no enhancing effect on that binding. This observation tends to validate the data of Lillis and collaborators and implies that C1q binding to LRP1 might have another biological role than that of a bridging molecule in a C1q/LRP1/CRT complex.

Overall our data indicate that similarly to many other LRP1 ligands, C1q binds to both clusters II and IV. The site responsible for LRP1 binding on C1q is located on its CLR, nearby the interaction site of the cognate protease tetramer C1r2s2. We propose a common canonical site for other C1q receptors and ligands, such as fucoidan and CR1. From a functional point of view, we show that C1q binds to both full-length and mini IV LRP1 receptors but that the first step of the uptake of late apoptotic cells by LRP1 is not influenced by C1q. Our results also highlight that cluster IV receptor can endorse most of LRP1 full-length binding capacities.

## Data Availability Statement

The datasets generated for this study are available on request to the corresponding authors.

## Author Contributions

JPK, VR, and NT designed the study. GF, EG, CW-P, IB, AC, JPK, and VR performed the research. GF, EG, CW-P, IB, JPK, VR, and NT analyzed the data. CDN and SD contributed key reagents. CG contributed to discussions and structural implements. VR, JPK, EG, and NT wrote the manuscript draft. All authors contributed to the article and approved the submitted version.

## Funding

This work has been supported by the French National Research Agency (grant ANR-16-CE11-0019) and by the University Grenoble Alpes (grant AGIR). This work used the platforms of the Grenoble Instruct-ERIC Center (ISBG; UMS 3518 CNRS CEA-UGA-EMBL) with support from the French Infrastructure for Integrated Structural Biology (FRISBI; ANR-10-INSB-05-02) and GRAL, a project of the University Grenoble Alpes graduate school (Ecoles Universitaires de Recherche) CBH-EUR-GS (ANR-17-EURE-0003) within the Grenoble Partnership for Structural Biology.

## Conflict of Interest

The authors declare that the research was conducted in the absence of any commercial or financial relationships that could be construed as a potential conflict of interest.

## Abbreviations

GR, globular region of C1q; CLR, collagen like region of C1q; CR, complement-type repeat; LDL, low-density lipoprotein; LRP1, LDL receptor-related protein 1; rC1qABC, recombinant C1q mutant LysA59/LysB61/LysC58, SP-A, surfactant protein A; SP-D, surfactant protein D; CRT, calreticulin; CR1, complement receptor1; MBL, mannose-binding lectin; MASP, MBL-associated serine protease; SPR, surface plasmon resonance; RAP, receptor-associated protein; RU, resonance unit; EGF, epidermal growth factor.

## References

[1] Galvan MD Greenlee-Wacker MC Bohlson SS C1q and phagocytosis: the perfect complement to a good meal. J Leukoc Biol (2012) 92:489–97. 10.1189/jlb.0212099 22715140

[2] Kouser L Madhukaran SP Shastri A Saraon A Ferluga J Al-Mozaini M Emerging and Novel Functions of Complement Protein C1q. Front Immunol (2015) 6:317. 10.3389/fimmu.2015.00317 26175731 PMC4484229

[3] Thielens NM Tedesco F Bohlson SS Gaboriaud C Tenner AJ C1q: A fresh look upon an old molecule. Mol Immunol (2017) 89:73–83. 10.1016/j.molimm.2017.05.025 28601358 PMC5582005

[4] Ogden CA deCathelineau A Hoffmann PR Bratton D Ghebrehiwet B Fadok VA C1q and Mannose Binding Lectin Engagement of Cell Surface Calreticulin and Cd91 Initiates Macropinocytosis and Uptake of Apoptotic Cells. J Exp Med (2001) 194:781–96. 10.1084/jem.194.6.781 PMC2195958 11560994

[5] Vandivier RW Ogden CA Fadok VA Hoffmann PR Brown KK Botto M Role of Surfactant Proteins A, D, and C1q in the Clearance of Apoptotic Cells In Vivo and In Vitro: Calreticulin and CD91 as a Common Collectin Receptor Complex. J Immunol (2002) 169:3978–86. 10.4049/jimmunol.169.7.3978 12244199

[6] Lillis AP Van Duyn LB Murphy-Ullrich JE Strickland DK LDL Receptor-Related Protein 1: Unique Tissue-Specific Functions Revealed by Selective Gene Knockout Studies. Physiol Rev (2008) 88:887–918. 10.1152/physrev.00033.2007 18626063 PMC2744109

[7] Duus K Hansen EW Tacnet P Frachet P Arlaud GJ Thielens NM Direct interaction between CD91 and C1q: Direct interaction between CD91 and C1q. FEBS J (2010) 277:3526–37. 10.1111/j.1742-4658.2010.07762.x 20716178

[8] Strickland DK Ranganathan S Diverse role of LDL receptor-related protein in the clearance of proteases and in signaling. J Thromb Haemost (2003) 1:1663–70. 10.1046/j.1538-7836.2003.00330.x 12871303

[9] Croy JE Shin WD Knauer MF Knauer DJ Komives EA All Three LDL Receptor Homology Regions of the LDL Receptor-Related Protein Bind Multiple Ligands ^†^. Biochemistry (2003) 42:13049–57. 10.1021/bi034752s 14596620

[10] Bu G Rennke S Receptor-associated protein is a folding chaperone for low density lipoprotein receptor-related protein. J Biol Chem (1996) 271:22218–24. 10.1074/jbc.271.36.22218 8703036

[11] Fisher C Beglova N Blacklow SC Structure of an LDLR-RAP Complex Reveals a General Mode for Ligand Recognition by Lipoprotein Receptors. Mol Cell (2006) 22:277–83. 10.1016/j.molcel.2006.02.021 16630895

[12] Arlaud GJ Sim RB Duplaa AM Colomb MG Differential elution of Clq, Clr and Cls from human Cl bound to immune aggregates. Use in the rapid purification of Cl subcomponents. Mol Immunol (1979) 16:445–50. 10.1016/0161-5890(79)90069-5 40870

[13] Tacnet-Delorme P Chevallier S Arlaud GJ -Amyloid Fibrils Activate the C1 Complex of Complement Under Physiological Conditions: Evidence for a Binding Site for A on the C1q Globular Regions. J Immunol (2001) 167:6374–81. 10.4049/jimmunol.167.11.6374 11714802

[14] Bally I Inforzato A Dalonneau F Stravalaci M Bottazzi B Gaboriaud C Interaction of C1q With Pentraxin 3 and IgM Revisited: Mutational Studies With Recombinant C1q Variants. Front Immunol (2019) 10:461. 10.3389/fimmu.2019.00461 30923526 PMC6426777

[15] De Nardis C Lössl P van den Biggelaar M Madoori PK Leloup N Mertens K Recombinant Expression of the Full-length Ectodomain of LDL Receptor-related Protein 1 (LRP1) Unravels pH-dependent Conformational Changes and the Stoichiometry of Binding with Receptor-associated Protein (RAP). J Biol Chem (2016) 292(3):912–24. 10.1074/jbc.M116.758862. jbc.M116.758862. PMC5247663 27956551

[16] Bally I Ancelet S Moriscot C Gonnet F Mantovani A Daniel R Expression of recombinant human complement C1q allows identification of the C1r/C1s-binding sites. Proc Natl Acad Sci (2013) 110:8650–5. 10.1073/pnas.1304894110 PMC3666734 23650384

[17] Jacquet M Cioci G Fouet G Bally I Thielens NM Gaboriaud C C1q and Mannose-Binding Lectin Interact with CR1 in the Same Region on CCP24-25 Modules. Front Immunol (2018) 9:453. 10.3389/fimmu.2018.00453 29563915 PMC5845983

[18] Arlaud GJ Thielens NM Human complement serine proteases C1r and C1s and their proenzymes. Methods Enzymol (1993) 223:61–82. 10.1016/0076-6879(93)23038-o 8271968

[19] Obermoeller-McCormick LM Li Y Osaka H FitzGerald DJ Schwartz AL Bu G Dissection of receptor folding and ligand-binding property with functional minireceptors of LDL receptor-related protein. J Cell Sci (2001) 114:899–908. 11181173 10.1242/jcs.114.5.899

[20] Jacquet M Lacroix M Ancelet S Gout E Gaboriaud C Thielens NM Deciphering Complement Receptor Type 1 Interactions with Recognition Proteins of the Lectin Complement Pathway. J Immunol (2013) 190:3721–31. 10.4049/jimmunol.1202451 23460739

[21] FitzGerald DJ Fryling CM Zdanovsky A Saelinger CB Kounnas M Winkles JA Pseudomonas Exotoxin-mediated Selection Yields Cells with Altered Expression of Low-Density Lipoprotein Receptor-related Protein.9. J Biol Chem (1995) 129(6):1533–41. 10.1083/jcb.129.6.1533 PMC2291175 7790352

[22] Bu G Williams S Strickland DK Schwartz AL Low density lipoprotein receptor-related protein/alpha 2-macroglobulin receptor is an hepatic receptor for tissue-type plasminogen activator. Proc Natl Acad Sci U S A (1992) 89:7427–31. 10.1073/pnas.89.16.7427 PMC49723 1502154

[23] Williams SE Ashcom JD Argraves WS Strickland DK A novel mechanism for controlling the activity of alpha 2-macroglobulin receptor/low density lipoprotein receptor-related protein. Multiple regulatory sites for 39-kDa receptor-associated protein. J Biol Chem (1992) 267:9035–40. 1374383

[24] Horn IR van den Berg BM van der Meijden PZ Pannekoek H van Zonneveld A-J Molecular Analysis of Ligand Binding to the Second Cluster of Complement-type Repeats of the Low Density Lipoprotein Receptor-related Protein Evidence for an allosteric component in receptor-associated protein-mediated inhibition of ligand binding. J Biol Chem (1997) 272:13608–13. 10.1074/jbc.272.21.13608 9153209

[25] Neels JG van den Berg BM Lookene A Olivecrona G Pannekoek H van Zonneveld A-J The second and fourth cluster of class A cysteine-rich repeats of the low density lipoprotein receptor-related protein share ligand-binding properties. J Biol Chem (1999) 274:31305–11. 10.1074/jbc.274.44.31305 10531329

[26] Sarafanov AG Makogonenko EM Andersen OM Mikhailenko IA Ananyeva NM Khrenov AV Localization of the low-density lipoprotein receptor-related protein regions involved in binding to the A2 domain of coagulation factor VIII. Thromb Haemost (2007) 98:1170–81. 10.1160/TH07-05-0353 18064310

[27] Willnow TE Orth K Herz J Molecular dissection of ligand binding sites on the low density lipoprotein receptor-related protein. J Biol Chem (1994) 269:15827–32. 7515061

[28] Moestrup SK Holtet TL Etzerodt M Thøgersen HC Nykjaer A Andreasen PA Alpha 2-macroglobulin-proteinase complexes, plasminogen activator inhibitor type-1-plasminogen activator complexes, and receptor-associated protein bind to a region of the alpha 2-macroglobulin receptor containing a cluster of eight complement-type repeats. J Biol Chem (1993) 268:13691–6. 7685767

[29] Downey GP Botelho RJ Butler JR Moltyaner Y Chien P Schreiber AD Phagosomal Maturation, Acidification, and Inhibition of Bacterial Growth in Nonphagocytic Cells Transfected with FcgRIIA Receptors.10. J Biol Chem (1999) 274:28436–44. 10.1074/jbc.274.40.28436 10497205

[30] Migliorini MM Behre EH Brew S Ingham KC Strickland DK Allosteric Modulation of Ligand Binding to Low Density Lipoprotein Receptor-related Protein by the Receptor-associated Protein Requires Critical Lysine Residues within Its Carboxyl-terminal Domain. J Biol Chem (2003) 278:17986–92. 10.1074/jbc.M212592200 12637503

[31] Nielsen KL Holtet TL Etzerodt M Moestrup SK Gliemann J Sottrup-Jensen L Identification of Residues in α-Macroglobulins Important for Binding to the α _2_ -Macroglobulin Receptor/Low Density Lipoprotein Receptor-related Protein. J Biol Chem (1996) 271:12909–12. 10.1074/jbc.271.22.12909 8662686

[32] Guttman M Prieto JH Handel TM Domaille PJ Komives EA Structure of the Minimal Interface Between ApoE and LRP. J Mol Biol (2010) 398:306–19. 10.1016/j.jmb.2010.03.022 PMC2880916 20303980

[33] Young PA Migliorini M Strickland DK Evidence That Factor VIII Forms a Bivalent Complex with the Low Density Lipoprotein (LDL) Receptor-related Protein 1 (LRP1):Identification of Cluster IV on LRP1 as the major binding site. J Biol Chem (2016) 291:26035–44. 10.1074/jbc.M116.754622 PMC5207074 27794518

[34] Duus K Thielens NM Lacroix M Tacnet P Frachet P Holmskov U CD91 interacts with mannan-binding lectin (MBL) through the MBL-associated serine protease-binding site: CD91 interacts with mannan-binding lectin. FEBS J (2010) 277:4956–64. 10.1111/j.1742-4658.2010.07901.x 21054788

[35] Garlatti V Chouquet A Lunardi T Vives R Paidassi H Lortat-Jacob H Cutting Edge: C1q Binds Deoxyribose and Heparan Sulfate through Neighboring Sites of Its Recognition Domain. J Immunol (2010) 185:808–12. 10.4049/jimmunol.1000184 20548024

[36] Almeda S Rosenberg RD Bing DH The binding properties of human complement component C1q. Interaction with mucopolysaccharides. J Biol Chem (1983) 258:785–91. 6218163

[37] Kirschfink M Blase L Engelmann S Schwartz-Albiez R Secreted chondroitin sulfate proteoglycan of human B cell lines binds to the complement protein C1q and inhibits complex formation of C1. J Immunol (1997) 158:1324–31. 9013976

[38] Tissot B Daniel R Place C Interaction of the C1 complex of Complement with sulfated polysaccharide and DNA probed by single molecule fluorescence microscopy. Eur J Biochem (2003) 270:4714–20. 10.1046/j.1432-1033.2003.03870.x 14622259

[39] Tissot B Montdargent B Chevolot L Varenne A Descroix S Gareil P Interaction of fucoidan with the proteins of the complement classical pathway. Biochim Biophys Acta BBA - Proteins Proteomics (2003) 1651:5–16. 10.1016/S1570-9639(03)00230-9 14499584

[40] Pflieger D Przybylski C Gonnet F Le Caer JP Lunardi T Arlaud GJ Analysis of Human C1q by Combined Bottom-up and Top-down Mass Spectrometry: detailed mapping of post-translational modifications and insights into C1r/C1s binding sites. Mol Cell Proteomics (2010) 9:593–610. 10.1074/mcp.M900350-MCP200 20008834 PMC2860232

[41] Jensen GA Andersen OM Bonvin AMJJ Bjerrum-Bohr I Etzerodt M Thøgersen HC Binding Site Structure of One LRP–RAP Complex:Implications for a Common Ligand–Receptor Binding Motif. J Mol Biol (2006) 362:700–16. 10.1016/j.jmb.2006.07.013 16938309

[42] Nikolic J Belot L Raux H Legrand P Gaudin Y A. Albertini A Structural basis for the recognition of LDL-receptor family members by VSV glycoprotein. Nat Commun (2018) 9:1029. 10.1038/s41467-018-03432-4 29531262 PMC5847621

[43] Dolmer K Campos A Gettins PGW Quantitative Dissection of the Binding Contributions of Ligand Lysines of the Receptor-associated Protein (RAP) to the Low Density Lipoprotein Receptor-related Protein (LRP1). J Biol Chem (2013) 288:24081–90. 10.1074/jbc.M113.473728 PMC3745351 23798683

[44] Migliorini M Li S-H Zhou A Emal CD Lawrence DA Strickland DK High-affinity binding of plasminogen-activator inhibitor 1 complexes to LDL receptor-related protein 1 requires lysines 80, 88, and 207. J Biol Chem (2020) 295:212–22. 10.1074/jbc.RA119.010449 PMC6952620 31792055

[45] Lillis AP Greenlee MC Mikhailenko I Pizzo SV Tenner AJ Strickland DK Murine Low-Density Lipoprotein Receptor-Related Protein 1 (LRP) Is Required for Phagocytosis of Targets Bearing LRP Ligands but Is Not Required for C1q-Triggered Enhancement of Phagocytosis. J Immunol (2008) 181:364–73. 10.4049/jimmunol.181.1.364 PMC2663906 18566402

