# Supplementary material for: Complement C1q Interacts With LRP1 Clusters II and IV Through a Site Close but Different From the Binding Site of Its C1r and C1s-Associated Proteases
Source: Front Immunol. 2020 Oct 21;11:583754. doi: 10.3389/fimmu.2020.583754 (PMC7609443; doi:10.3389/fimmu.2020.583754)
Supplement: Supplementary file 1 [file DataSheet_1.pdf]

Supplementary Material

Complement C1q interacts with LRP1 clusters II and IV through a site close but different from the binding site of its C1r and C1s associated proteases

Guillaume Fouët[1†], Evelyne Gout[1†], Catherine Wicker-Planquart[1], Isabelle Bally[1], Camilla de Nardis[2], Stéphane Dedieu[3], Anne Chouquet[1], Christine Gaboriaud[1,] Nicole M. Thielens[1], Jean Philippe Kleman[1*], Véronique Rossi[1*].

***Correspondence:**
Corresponding Authors
veronique.rossi@ibs.fr; jean-philippe.kleman@ibs.fr

Supplementary Figures S1, S3 and S3

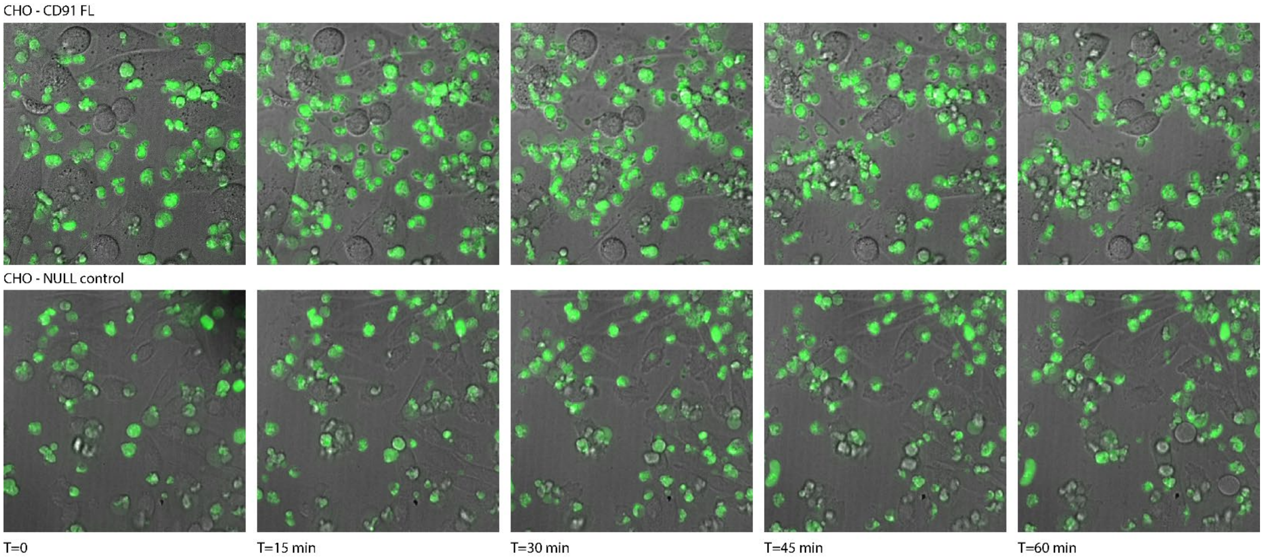

Supplementary S1 – Time Lapse Immunofluorescence analysis of the interaction of apoptotic Jurkat cells with CHO expressing full-length LRP1

Control NULL and LRP1 FL CHO cells were plated in 35mm glass bottom dishes the day before analysis. Labeled apoptotic cells (green fluorescence) were added in the medium over the spread CHO cells (visible in the transmission light channel, gray) and subsequently imaged every 3 minutes using spinning disk confocal microscopy, maintaining the 488 excitation laser at a minimal value of 1% of its nominal power. The figure (1 timepoint shown every 15 min) illustrates the typical redistribution of the apoptotic cells around the CHO LRP1 FL cells but not around control NULL cells.

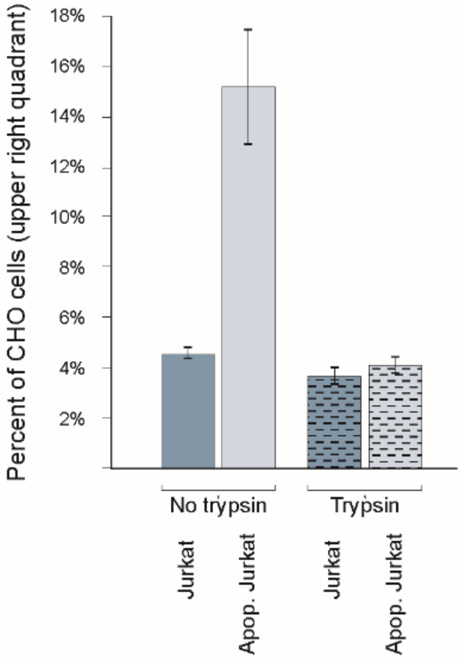

Supplementary S2 – Trypsin experiment

Flow cytometry analysis after trypsin treatment of CHO cells in contact with healthy or apoptotic Jurkat cells. CHO cells expressing full-length LRP1 were incubated with Jurkat cells before being harvested and post-treated by trypsin.

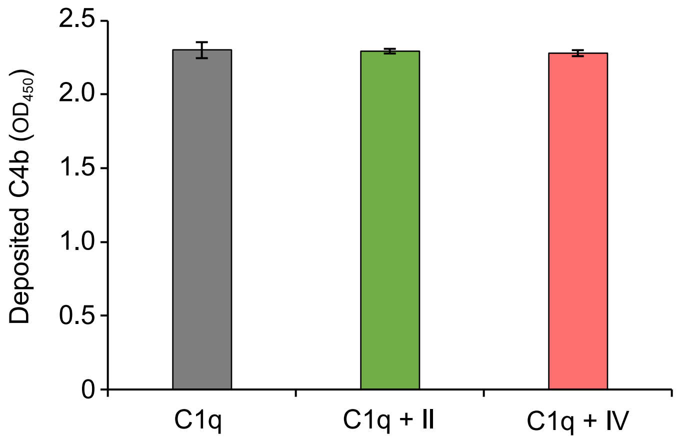

Supplementary S3 – Complement activation in the presence of LRP1 clusters II and IV

C1q-depleted serum (CompTech, 1:25 dilution) was reconstituted with purified serum C1q (4 µg/mL) or C1q (4 µg/mL) that had been preincubated 15 min at room temperature with cluster II or IV at a 100-fold molar excess. This mixture was added to microwells coated with 2 µg/mL IgM. The resulting C1-cleaving activity was measured by C4b deposition assay as described in (14). Deposited C4b was detected with an anti-human polyclonal antibody, and results are expressed as absorbance at 450 nm (OD_{450}), following background subtraction [mean ± SEM of two and three independent experiments for cluster II and IV respectively]. Normal human serum was used as a positive control (OD_{450} = 2.32 ± 0.03).
